# Is consonance attractive to budgerigars? No evidence from a place preference study

**DOI:** 10.1007/s10071-020-01404-0

**Published:** 2020-06-22

**Authors:** Bernhard Wagner, Daniel L. Bowling, Marisa Hoeschele

**Affiliations:** 1 Acoustics Research Institute, Wohllebengasse 12-14, 1040 Vienna, Austria; 2 Department of Psychiatry and Behavioral Sciences, Stanford University School of Medicine, 1201 Welch Rd. MSLS P-126, Stanford, CA 94305-5485 USA; 3 Department of Cognitive Biology, Althanstrasse 14 (UZA1), 1090 Vienna, Austria

**Keywords:** Consonance, Budgerigars, Psychoacoustics, Comparative cognition, Aesthetics, Vocal learning

## Abstract

Consonant tone combinations occur naturally in the overtone series of harmonic sounds. These include sounds that many non-human animals produce to communicate. As such, non-human animals may be attracted to consonant intervals, interpreting them, e.g., as a feature of important social stimuli. There is preliminary evidence of attraction to consonance in various bird species in the wild, but few experimental studies with birds. We tested budgerigars (*Melopsittacus undulatus*) for attraction to consonant over dissonant intervals in two experiments. In Experiment 1, we tested humans and budgerigars using a place preference paradigm in which individuals could explore an environment with multiple sound sources. Both species were tested with consonant and dissonant versions of a previously studied piano melody, and we recorded time spent with each stimulus as a measure of attraction. Human females spent more time with consonant than dissonant stimuli in this experiment, but human males spent equal time with both consonant and dissonant stimuli. Neither male nor female budgerigars spent more time with either stimulus type. In Experiment 2, we tested budgerigars with more ecologically relevant stimuli comprised of sampled budgerigar vocalizations arranged into consonant or dissonant chords. These stimuli, however, also failed to produce any evidence of preference in budgerigar responses. We discuss these results in the context of ongoing research on the study of consonance as a potential general feature of auditory perception in animals with harmonic vocalizations, with respect to similarities and differences between human and budgerigar vocal behaviour, and future methodological directions.

## Introduction

Combinations of pitches (i.e., the percept of fundamental frequency) related by simple integer ratios tend to be perceived as relatively pleasant to humans, as was already observed in ancient Greece by Pythagoras (Crocker 1963) as well as in ancient China (Chen 1996). These combinations are referred to as consonant, while other, perceptually less attractive combinations are referred to as dissonant (see e.g. Bowling and Purves 2015; Krumhansl 1990; Terhardt 1984). This human preference for consonant over dissonant intervals correlates with a preference for harmonic sounds such as those found in the human voice (Bowling et al. 2018; Cousineau et al. 2012; McDermott et al. 2010). This relationship may derive from the fact that the most energetic harmonics (i.e., the ones that are closest to the fundamental) in the series produced by vocal fold vibration form all of what are known as “perfect” intervals in Western music theory. With respect to the fundamental (i.e., the first harmonic), the second harmonic is a perfect octave (2:1), the third harmonic is a perfect fifth (3:2) above the second, and the fourth harmonic, a perfect fourth above the third (4:3). These intervals are considered consonant by listeners from a variety of cultures and are found throughout music from around the world (Burns 1999). These findings have led to the hypothesis that our attraction to consonant tone combinations is based in part on the biology of vocal communication (e.g., Bowling and Purves 2015; Schwartz et al. 2003; Terhardt 1984). We will refer to the hypothesis that an attraction to harmonic vocalizations constitutes the biological foundation of consonance preferences as the “vocal similarity hypothesis” (“VSH”; Bowling and Purves, 2015; Bowling et al., 2018).

For humans, a preference for consonance appears to develop early in life: infants prefer listening to consonant over dissonant intervals from 2 months of age (Trainor et al. 2002). Newborn babies show activation of different brain hemispheres when hearing consonance or dissonance (Perani et al. 2010). Infants as young as 6 months show more sensitivity to tuning changes in consonant than in dissonant interval sequences (Schellenberg and Trehub 1996). Infants also move less and show more attention and positive affect for consonant stimuli (Trainor and Heinmiller 1998; Trehub 2003; Zentner and Kagan 1996, 1998). This is true even for newborns regardless of whether or not their parents are deaf (Masataka 2006). However, Platinga and Trehub (2014) have challenged some of these results, arguing that familiarity is the key determining factor in the measures of preference used in these infant studies. For adults, musical training also impacts consonance perception, sharpening the contrast between intervals considered consonant and dissonant in music theory for musicians compared to non-musicians (see e.g. Bowling et al. 2018; McDermott et al. 2010). Thus, the basis of human consonance preference remains debated (see e.g. Bowling et al. 2017; McDermott et al. 2016; Virtala and Tervaniemi 2016).

One way to study consonance perception in the absence of cultural and familiarity confounds is to test preferences in non-human animals where prior exposure to human music (which is largely based on consonance) can be controlled. Cross-species comparisons can be used to assess the degree to which consonance preferences are a general phenomenon, and the degree to which they may be constrained by acoustic details of species-specific auditory–vocal communication. However, research in this direction remains limited with only a handful of species having been studied, using variable paradigms. Some studies have tested not for preference but for the ability to discriminate between consonant and dissonant stimuli. Such studies found that Japanese macaques (Izumi 2000) as well as European starlings and Java sparrows were able to discriminate between consonant and dissonant stimuli (Hulse et al. 1995; Watanabe et al. 2005), clearly a prerequisite but no definitive sign for consonance preference. Several studies have applied more direct measures of preference for consonance. For Tungara frogs, manipulation of frequency ratio in mating calls to produce consonant or dissonant intervals had no influence on female attraction (Akre et al. 2014). Albino rats showed preference for consonant stimuli in an early study (Fannin and Braud 1971), but in a recent study, brown rats only learned to discriminate between consonant and dissonant chord stimuli that they had been trained on, failing to generalize to novel consonant and dissonant stimuli (Crespo-Bojorque and Toro 2015). No preference for consonance or dissonance was observed in cotton-top tamarins (McDermott and Hauser 2004) as well as in Campbell’s monkeys (Koda et al. 2013). An infant chimpanzee was shown to prefer consonant intervals (Sugimoto et al. 2010). However, the chimpanzee’s early auditory environment was not controlled, and given that it was hand raised by humans, may likely have been exposed to music prior to test as pointed out by Chiandetti and Vallortigara (2011). For birds, research is even sparser, but has yielded results suggesting that some species are attracted to consonance. Musician wren and hermit thrush songs contain harmonic interval sequences (Doolittle and Brumm 2012; Doolittle et al. 2014) and for great tits, production of harmonic intervals has been shown to be related to mating success (Richner 2016) which may hint at a preference for consonance at least in this context. We are aware of only one published experimental study that tested for attraction to consonance in birds. This was performed with a paradigm testing chicks (*Gallus domesticus*) newly hatched from eggs incubated in acoustic isolation (Chiandetti and Vallortigara 2011). These chicks were shown to preferentially approach imprinting objects spacially associated with consonant rather than dissonant music, thus providing evidence that domestic chickens exhibit an attraction to consonance that is not dependent on prior exposure to music. The authors argued that the chicks may have used consonance as an indicator of the object likely to be their mother, who would produce harmonic sounds.

In addition to providing young animals with a cue towards their parents, we hypothesized that vocal learning may be another context in which exhibiting a perceptual attraction to harmonic sounds would be beneficial. An animal that must learn its vocalizations from a tutor needs to pay special attention to the tutor’s vocalizations, which in many cases are differentiated from other environmental sounds by harmonic structure. If this were the case, an attraction to consonance should be more common for vocal learning species. Previous experimental studies of consonance in non-human animals have used non-vocal learning mammals and birds. The potential role of vocal learning in shaping consonance perception thus remains entirely unexplored.

Here, we tested budgerigars (*Melopsittacus undulatus*), a small vocal learning parrot species endemic to Australia (Tu et al. 2011; Tyack 2008) for attraction to consonance over dissonance using a place preference paradigm. Budgerigar vocal learning abilities are life long with males imitating female mates (Hile et al. 2005), converging calls within groups (Hile et al. 2000; Hile and Striedter 2000) and occurences of mimicry of non species-specific sounds such as human speech (Gramza 1970; Scanlan 1999). As such, we tested adult birds. We also tested adult humans in a parallel paradigm. To our knowledge, this study represents the second experimental study to assess attraction to consonance in a bird species and the first study to do so in a non-human vocal learner.

## Experiment 1

In this experiment, we tested budgerigars and adult humans. To facilitate comparison with previous studies, we used the same stimuli that were used to test newly hatched chicks (Chiandetti and Vallortigara 2011) and infants (Zentner and Kagan 1998). These were consonant and dissonant versions of two piano melodies. We tested humans using the same paradigm and stimuli to test if this approach is sufficient to measure consonance preferences.

### Methods

#### Compliance with ethical standards

All applicable international, national, and/or institutional guidelines for the care and use of animals were followed. All procedures performed in studies involving human participants were approved by the University of Vienna Ethics Committee (Approval Number 00063) and were conducted in line with the declaration of Helsinki (1964). Participants filled out an informed consent form, which included the provision that they could withdraw from the study at any time without further consequences. All procedures performed in studies involving animals were in accordance with Austrian animal protection and housing laws and were approved by the ethical board of the behavioural research group in the faculty of Life Sciences at the University of Vienna (Approval Number 2015–005).

#### Human subjects

We tested 20 adult humans (10 male, 10 female) at the University of Vienna. They were aged between 18 and 32 years (mean = 22.7, SD = 3.5). These participants were recruited either directly by an assistant researcher, or via an online service system (Sona Systems; Tallinn, Estonia) where potential participants were registered and could sign up for experiments for monetary compensation. The majority of the participants were students at the University of Vienna. None of the participants had any prior knowledge about the experiment. The subjects had taken weekly music lessons for 4.8 years on average (SD = 3.3; range = 0–13). The amount of experience was not significantly different between males and females (male mean = 4.1, female mean = 5.5, Mann–Whitney *U* = 41.5; *P* = 0.5418).

#### Budgerigar subjects

We tested a total of 12 adult budgerigars (6 male, 6 female) aged between 1 and 4 years (sexual maturity reached within first 4–8 months; Brockway 1964), that were housed at the Animal Care Facility in the Department of Cognitive Biology at the University of Vienna maintained on a 12.5/11.5 h light/dark cycle, approximating budgerigar’s diurnal cycle (Wyndham 1980). The birds were kept in three aviaries (2 × 1 × 2 m) containing groups of five to ten budgerigars. Nine of the budgerigars had prior experience with the testing apparatus, but all were naive to the current stimulus set. The birds were kept on a diet of water (ad libitum) and pellet bird food with vitamin supplements.

#### Apparatus for human testing

**Fig. 1:**
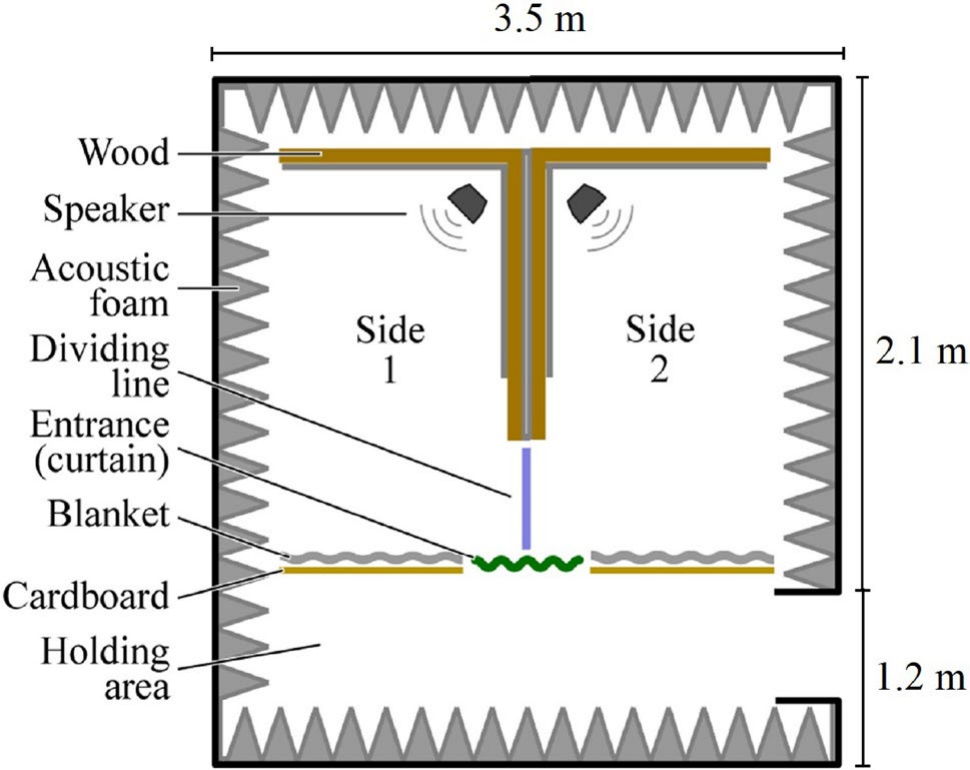
Testing apparatus for humans. 3.2 m high. The dividing blue line marks cut-off used when coding video data to determine subjects’ location at any time. The outer walls, ceiling, and floor of the chamber were the walls of the anechoic room, except for the entrance wall, which was made out of large cardboard sheets and heavy blankets. The bisecting wall was made out of heavy sheets of wood, cardboard, and blankets (Figure adapted from Hoeschele and Bowling 2016)

Figure 1 shows a diagram of the place preference chamber used to test humans. A large rectangular room was divided into two sides by a bisecting wall as described by Hoeschele and Bowling (2016). The left and right side were identical: they were lit by an overhead lighting fixture and contained a single speaker (M-Audio AV 40, Cumberland, RI, USA) placed at the end opposite the entrance to the chamber. Otherwise they were empty. The entrance to both sides was in the middle of one of the long sides of the rectangle, making sure that participants had to choose immediately whether to go to the left or right side once they entered the room. The human testing chamber was constructed within an anechoic room. This was done to reduce transmission of sound from one side of the chamber to the other. To block visual access between this holding area and the room, a curtain was hung over the entrance itself. Movement within the setup was recorded by two overhead cameras (C920 HD Pro Webcam; Logitech, Lausanne, Switzerland), each recording one side only.

#### Apparatus for budgerigar testing

**Fig. 2:**
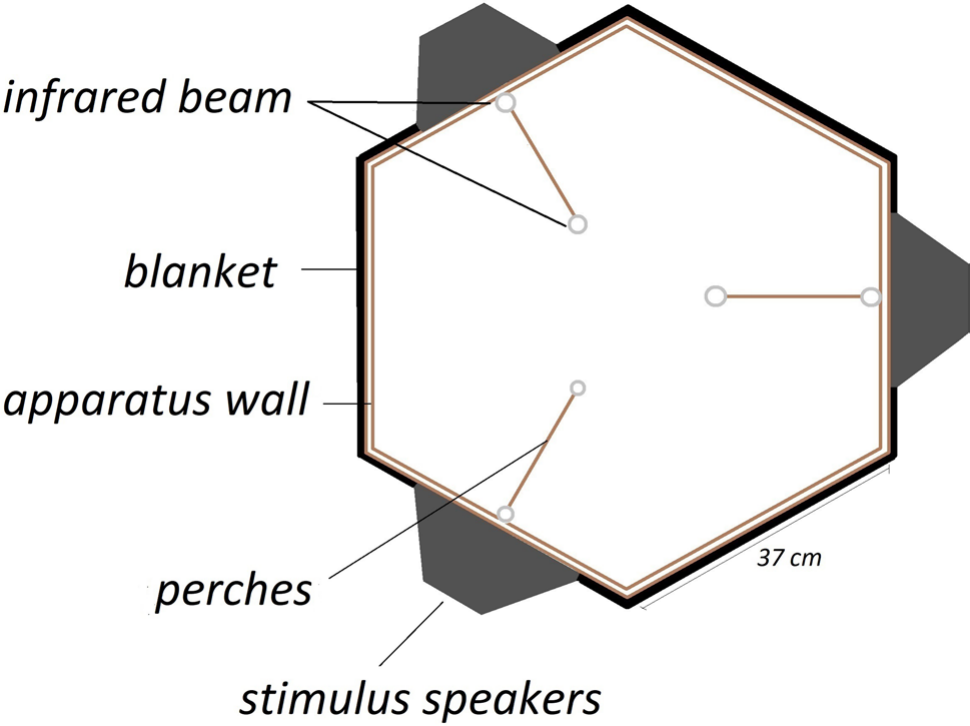
Budgerigar testing apparatus top view. Three perches are marked inside the apparatus 70 cm above apparatus floor. Each perch contained an infrared beam that monitored the bird’s position and was positioned next to a stimulus speaker. The outer walls were made of wire mesh and wood. A black encircling blanket was used to keep all sides visually identical. Height of the apparatus: 1.1 m. The apparatus was lit by room lights through translucent roof. A background noise speaker was positioned above the roof

Figure 2 shows a diagram of the place preference testing apparatus used to test budgerigars. The budgerigar testing took place in a hexagonal testing aviary. The apparatus contained three perches each of which had one of three speakers (FE108 full-range speaker, Fostex, Tokyo, Japan; frequency range 200–16 000 Hz) positioned next to it. A bird landing on one of the perches was registered by disruption of an infrared beam (IR Break Beam Sensor—3 mm LEDs, Adafruit Industries, New York, NY, USA). A disruption of the infrared beam for more than 300 ms triggered the playback of stimuli via the adjacent speaker from a MacBook Pro (Apple Inc., Cupertino, CA, USA) Arduino Uno REV 3 chip (BCMI, Turin, Italy). This functionality was programmed in “Python”. Pilot testing showed that birds would cling to the side of the cage and not interact with the apparatus if they were in acoustic isolation from their flock. Therefore, colony playback was implemented. Above the apparatus, a speaker (M-Audio AV 40, Cumberland, RI, USA) controlled by an iPad (Apple Inc., Cupertino, CA, USA) administered wav-sound files (> 2 h 15 min) recorded from the bird’s home aviary presented at ~ 50 dB at the position of the bird to reduce the stress of isolation in the test subjects. A food bowl and a water bowl were placed in the center of the aviary floor during testing.

### Procedure

#### Human testing

Participants were informed that the experiment consisted of entering a testing chamber with sounds being played at a comfortable level. They were instructed that they could enter the chamber through the entrance curtain as soon as they heard sound playing inside and were told that they could freely explore the space inside as they pleased. They were also told to exit the chamber when the sounds ended. Once the participant was ready, the experiment followed a three step procedure (Hoeschele and Bowling 2016): (1) The experimenter initiated video recording and acoustic playback from the speakers via a computer (an Apple Mac Mini playing a single stereo track with different stimuli on the left and right channels; side order counterbalanced across participants); (2) participants were permitted to enter the chamber and their movements were recorded and (3) after 5 min, playback was ended and the participant exited the chamber. After completion of their time in the chamber, participants were asked to complete a brief computerized survey (LiveCode Community 7.0.5; Edinburgh, Scotland) in which they listened to each stimulus again. The survey asked participants to rate the two versions of the stimuli on visual analogue scales (later converted to an integer between − s100 and 100) with regard to their personal preference towards the stimuli and their interest in them. The video data (without audio) were blind coded to calculate the proportion of time participants spent on each side of the experimental chamber. Proportion of time was used as a measure of the participant’s preference for the corresponding stimulus. Human participants were coded as being on either side of the chamber if both of their feet were fully on one side of the bisecting wall (see dividing line in Fig. 1). In all other cases, the location was coded as being on neither side. We excluded the time that participants were on neither side from final analysis.

#### Budgerigar testing

Budgerigars were tested in multiple sessions. Individual budgerigars were taken from their home aviaries to a different room containing the testing apparatus described above. Birds were then placed in the apparatus and left alone to move freely within it for the duration of two hours. On one perch, the birds heard consonant stimuli, on another dissonant stimuli. The third perch was designated as silent. The positions of the stimuli were counterbalanced across birds and across sessions to control for perch biases. If a bird stayed on a perch for longer than the duration of a stimulus wav-file another stimulus would play. If a bird left a perch before the stimulus finished playing the stimulus wav-file would end (5 ms linear fade out). The program cycled through all stimuli in random order without replacement until all stimuli had been heard and then cycled through them again in random order without replacement. Leaving a perch and then returning to it would initiate playback of a new stimulus. The program recorded: (1) actual time on each perch; (2) stimulus time for each perch; (3) landings per perch and (4) landings per perch ≥ 300 ms (the time it took to trigger stimulus playing). After completion of a 2 h session, birds were taken back to their home aviary. This procedure was repeated on subsequent days until birds had completed at least three sessions. We chose not to implement a pre-training familiarization phase, because it is established that familiarization can drive preferences in humans (Bowling et al. 2018; McDermott et al. 2010; see also Platinga and Trehub 2014). Thus, it was important to our purpose that we would record the budgerigars’ spontaneous reaction to novel stimuli. For this reason, we also had to set a criterion of which data to analyse: if a subject had not heard both a consonant and a dissonant stimulus over the first three sessions at least once (as occurred for one bird), they received further sessions up to a maximum of six sessions in total. If a subject had not heard both a consonant and a dissonant stimulus by the sixth session they were excluded from the experiment (as occurred for one bird). To control for a potential effect of exposure to the stimuli, we conducted additional data analysis including only data from the point where birds had heard all stimulus types at least once.

#### Stimuli

In this experiment, the same stimuli were used to test humans and budgerigars. These stimuli were consonant and dissonant versions of two melodies, A and B produced in the music notation program “Sibelius” (Avid Technology Inc., Burlington, M, USA) using a piano midi timbre. These melodies used the same note progression as the ones used to test newly hatched chicks (Chiandetti and Vallortigara 2011) and infants (Zentner and Kagan 1998). Each melody repeated for 5 min and 10 s and contained notes from F3 (174.61 Hz) to C#5 (554.37 Hz). Consonant versions consisted of a sequence of vertically stacked major 3rds and perfect 5ths, dissonant versions consisted of sequences of vertically stacked minor 2nds and major 7ths. These melodies were rated as pleasant (consonant) and unpleasant (dissonant), respectively, by 23 out of 24 listeners in Zentner and Kagan (1998). Melody B used the same tone intervals in a different order. The melodies were presented at 75 dB for budgerigars and for humans. All these frequencies are well within budgerigar and human hearing range at the amplitudes used here (see Heffner et al. 2016; Okanoya and Dooling 1987) and have been successfully identified by budgerigars in previous operant work (see Dent et al. 2000; Dooling et al. 1995; Wagner et al. 2019; Weisman et al. 2004).

#### Analysis

Statistical analysis of the data was performed in “R” (R Core Team, GNU General Public License v2) using “Rstudio” (RStudio, Inc., Affero General Public License). As the data could not be assumed to be normally distributed for humans (Shapiro–Wilk, *p* = 0.00012), we used the Wilcoxon signed-rank test (a non-parametric alternative to the *t* test appropriate for paired samples and repeated measurements) to determine whether there was a significant difference in the time spent with consonant and dissonant stimuli as well as the preference and interest ratings given for consonant and dissonant stimuli, respectively. Sex differences in budgerigar acoustic preferences were shown in a previous place preference study (Hoeschele and Bowling 2016). For humans, sex differences in acoustic perception have sometimes been found (Bowling et al. 2019; Nater et al. 2006; Wuttke-Linnemann 2019), and while sex differences in human consonance perception have not been reported, it is not clear that they have been investigated. For these reasons and for parallelity, we analysed the data for each sex separately as well in both humans and budgerigars using the same tests as described above. To analyse results from the questionnaire, Spearman correlations were used to assess correlations between difference in stimuli ratings given by participants and difference in time spent with the differing stimuli.

The budgerigar data also showed a trend towards non-normal distribution (Shapiro–Wilk test, *p* = 0.09595). With small sample sizes like the ones in this study, normality tests are not always reliable (Ghasemi and Zahediasl 2012). We, therefore, deemed it better to use non-parametric tests (as above) that also have high efficiency with normal distributed data, and not risk violating the assumption of normality required for a *t* test. A Wilcoxon signed-rank test was used to determine whether budgerigars spent differing amounts of time with consonant and dissonant stimuli. A Friedman test (a non-parametric alternative to the ANOVA for non-parametric repeated measures data) was used to determine whether budgerigars spent significantly more time in one out of the three stimulus conditions. We also analysed the data for each sex separately as described above.

### Results

#### Human testing results

The video data from the experiment were blind coded, with 8 out of 20 sessions being coded twice to test inter-rater reliability, which was found to be at 97.7%. One male participant exited the testing chamber before their 5 min session was complete and was, therefore, excluded from analysis. One male and one female participant spent the entire time on only the consonant stimulus side. These participants were also excluded as their relative preferences could not be evaluated.

**Fig. 3:**
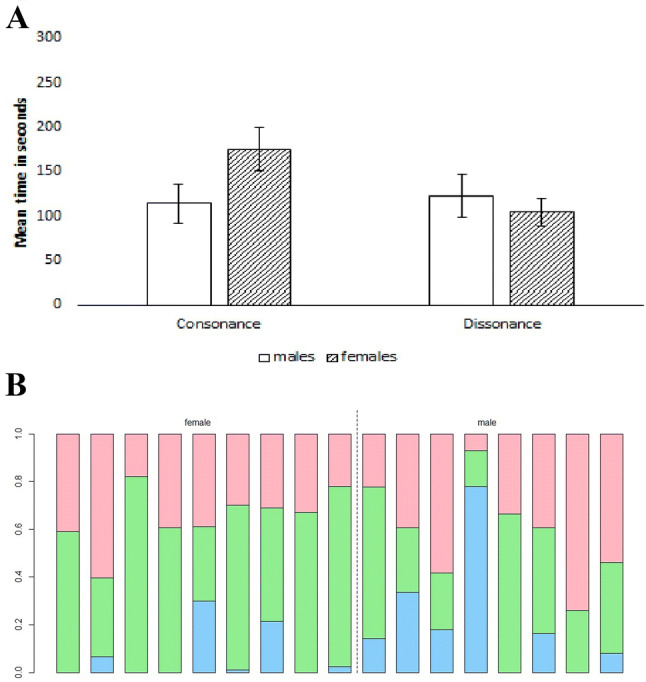
**a** Mean average time spent with consonant and dissonant stimuli for human participants seperated for males and females. Error bars show standard error. **b** Relative time spent with consonant and dissonant stimuli for human participants. Green is time on consonant (middle), red is time on dissonant (top), blue is time spent in neither condition (excluded from final analysis; bottom). Females are to the left of the dividing line; males are to the right (color figure online)

Figure 3a gives the mean average and standard error for time spent with the different stimulus types and silence for males and females, respectively. See Fig. 3b for a visual representation of relative time spent with consonant stimuli, dissonant stimuli and on neither side for individuals.

Using a Wilcoxon signed-rank test across all participants, we identified a trend towards spending more time with consonant stimuli (*M* = 2 min 26 s, SD = 1 min 7 s) than with dissonant (*M* = 1 min 53 s, SD = 54 s) stimuli (Wilcoxon, *p* = 0.08862). We then conducted a Wilcoxon signed-rank test to compare time spent with consonant and dissonant stimuli separately for each sex revealing that females spent significantly more time with consonant (*M* = 2 min and 55 s, SD = 1 min 11 s) than with dissonant stimuli (*M* = 1 min 44 s, SD = 46 s; Wilcoxon, *p* = 0.02734). Males, however, did not spend significantly more time with either consonant (*M* = 1 min 54 s, SD = 1 min 1 s) or dissonant stimuli (*M* = 2 min 3 s, SD = 1 min 8 s; Wilcoxon, *p* = 0.6797).

We used a Wilcoxon signed-rank test to compare ratings for the stimuli as given by participants in the questionnaire. Significantly higher preference ratings for consonant stimuli over dissonant stimuli were given by females (Wilcoxon, *p* = 0.007133) as well as males (Wilcoxon, *p* = 0.01953). Significantly higher ratings of interest were given by males for dissonant stimuli over consonant stimuli (Wilcoxon, *p* = 0.01125), while there was no significant difference in interest ratings given by females (Wilcoxon, *p* = 0.3834).

We used Spearman correlations to analyse the ratings for stimuli given by participants in the questionnaire and the relative time they spent with consonant and dissonant stimuli. We correlated the difference between relative time spent with consonant and dissonant stimuli with the difference between the ratings for consonant and dissonant stimuli. There was a significant correlation for the difference between relative time spent with consonant and dissonant stimuli and the difference between the preference ratings for these stimuli for females (Spearman, rho = 0.7666667, *p* = 0.02139) but not for males (Spearman, rho = 0.4285714, *p* = 0.2992). There was a significant correlation for the difference between relative time spent with consonant and dissonant stimuli and the difference between interest ratings for males (Spearman, rho = 0.7380952, *p* = 0.04583) but not for females (Spearman, rho = − 0.2833333, *p* = 0.463).

#### Budgerigar testing results

**Fig. 4:**
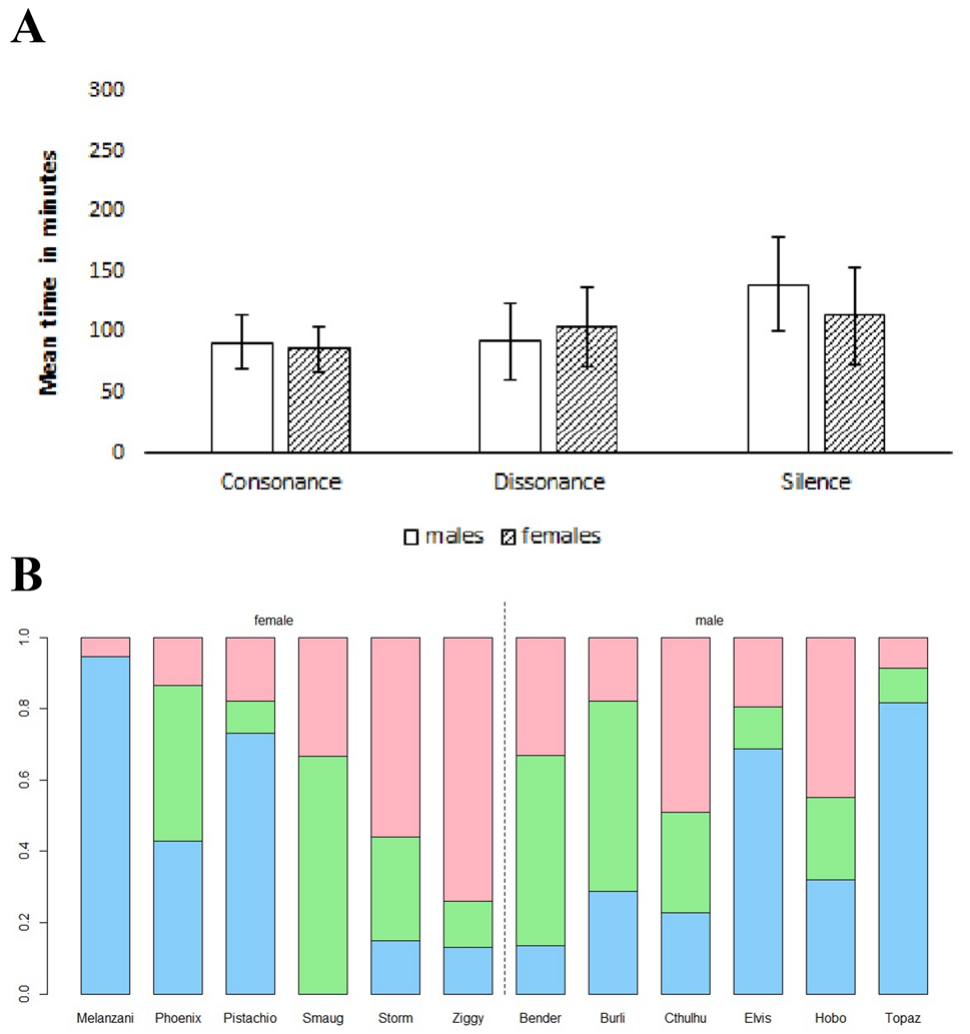
**a** Mean time spent with consonant and dissonant stimuli for budgerigars separated by males and females. Error bars show standard error. **b** Relative time spent with consonant and dissonant stimuli for budgerigar subjects. Green is time on consonant (middle), red is time on dissonant (top), blue is time on silent (bottom). Females are to the left of the dividing line, males are to the right (color figure online)

Figure 4a gives the mean average and standard error for time spent with the different stimulus types and silence for males and females, respectively. See Fig. 4b for a visual representation of relative time spent with consonant stimuli, dissonant stimuli and on neither side for individuals.

We used a Friedman test to compare relative time spent on the consonant (*M* = 89 min 18 s, SD = 67 min 35 s), dissonant (*M* = 98 min 36 s, SD = 64 min 59 s) and silent condition (*M* = 126 min 41 s, SD = 96 min 59 s). Budgerigars did not spend significantly more time on any either of these conditions in this experiment (Friedman, *p* = 0.92).

Additional Wilcoxon signed-rank tests analysing each sex separately showed that, for males, there was no significant difference in time spent with consonant (*M* = 92 min 24 s, SD = 52 min 35 s) or dissonant (*M* = 92 min 28 s, SD = 46 min 26 s) stimuli (Wilcoxon, *p* = 0.5). The same is true for females: there was no siginificant difference between time spent with consonant (*M* = 86 min 12 s, SD = 79 min 42 s) and dissonant (*M* = 92 min 28 s, SD = 78 min 50 s) stimuli (Wilcoxon, p = 0.7187).

To control for an effect of exposure, we also analysed data only from beyond the point where birds had heard each stimulus at least once. We found no diverging results with all *p* values > 0.05 (for all birds, Wilxocon *p* = 0.6177; for males only Wilcoxon *p* = 0.1563; for females only Wilcoxon *p* = 0.9062).

#### Summary

This experiment used piano stimuli originally designed for humans (Zentner and Kagan 1998) to test if humans and budgerigars would spend more time with consonant stimuli in a place preference paradigm. Human females spent significantly more time with the consonant stimuli, but human males did not, providing the first evidence of sex differences that we are aware of, relevant to consonance perception in humans. Budgerigars, however, did not spend significantly more time with either consonant or dissonant stimuli, regardless of sex. While this result supports the conclusion that budgerigars do not exhibit an attraction to consonance it may also have been due to the experimental stimuli being human specific. The stimuli used here were played using a MIDI-piano setting in a pitch range typical of human speech and music. Budgerigar vocal communication, however, differs markedly from these stimuli in pitch, timbre and temporal dynamics (Hile et al. 2000; Lavenex 1999; Tu et al. 2011). While the stimuli used here were clearly in budgerigar’s hearing range, it has been documented that their hearing is most accurate in the range of their own vocalizations (Heffner et al. 2016; Okanoya and Dooling 1987). Similarly, timbre and temporal dynamics more similar to budgerigars’ vocalizations could conceivably be more salient to their perception. To address the possibility that results would differ with stimuli catered to species-specific perception, we conducted a second experiment.

## Experiment 2

To examine the possibility that the budgerigar results from Experiment 1 reflect the low salience of stimuli with little ecological relevance, we conducted a second budgerigar experiment with novel stimuli specifically composed for budgerigars. These stimuli consisted of recorded budgerigar vocalizations, manipulated to change their pitch and then combined into chords typically perceived as either consonant or dissonant by humans.

### Methods

Experiment 2 was conducted with budgerigars only because pitch range (e.g. Oxenham 2012) and speed of the budgerigar stimuli (Dent et al. 2000) would make it difficult for human participants to discriminate the two sets of stimuli. With the exception of the stimuli, the methods were the same as in Experiment 1. The individual budgerigars tested were also the same as in Experiment 1, except for three birds that had to be replaced due to attrition. These individuals were replaced with budgerigars of the same sex. Two of the replaced birds were male, one was female. The female bird had previous experience with the apparatus but was naïve to the used stimulus set, the male birds were naïve to both. Five months elapsed between Experiments 1 and 2.

#### Stimuli

The stimuli for this experiment were WAV files created in “Matlab” (The MathWorks, Inc., Natick, Massachusetts, USA) using custom code written for the purpose of this experiment. They consisted of harmonic segments of budgerigar warble song recorded from a single bird (to which the experimental subjects had no previous contact or exposure), pitch shifted and combined to generate chords, that is, simultaneous occurrences of multiple such sounds. The budgerigar from which the vocalizations were sampled was recorded in a home in Arkansas, USA. It was housed with another budgerigar in a metal wire cage (70 × 60 × 50 cm) and was recorded with an H4N Zoom recorder and a Sennheiser directional shotgun microphone at a sampling rate of 44.1 kHz, 16 bit. Their cage was lined with acoustic foam padding (Basotect® 30 Plain; BASF Global) to reduce outside noise and echo. The recordings were made after habituating these birds to the presence of recording equipment in their social environment to be able to record as naturalistic sounds as possible. A total of 12 vocalization segments were sampled, having been selected and extracted from a longer recording based on the presence of relatively rich harmonic spectra. The F0s of these vocalizations prior to pitch shifting ranged from 495–1412 Hz (mean = 1117 Hz, SD = 251 Hz). Duration ranged from 44 to 174 ms (mean = 84 ms, SD = 39 ms). The chords into which these vocalizations were combined were selected on the basis of their being reliably perceived as highly consonant or dissonant by human raters in a recent study (Bowling et al. 2018). For each instance of a given chord, one vocalization was selected and pitch shifted to create each component tone, with F0 relationships defined by just intonation ratios (as given in Bowling et al. 2018) and such that the mean F0 of all component tones was maintained at 1130 Hz (the median F0 of all 12 vocalization samples). There were 44 stimuli in total, 22 consonant and 22 dissonant. Each stimulus lasted for 1 min and featured a single chord every 50 ms, each being built out of one of the 12 vocalizations, selected at random without replacement until all 12 vocalizations were used, and then repeated for the next set of 12 chords. Stimuli were presented at ~ 75 dB as measured at the average listening position of a bird on a perch (Table 1).

**Table 1:** Description of selected consonant and dissonant chords for Experiment 2

Chord type	Name	Tone 2	Tone 3	Tone 4
Consonant chords				
Dyad	Octave	12	–	–
Dyad	Perfect 5th	7	–	–
Triad	Minor triad (r)	3	7	–
Triad	Major triad (1st)	3	8	–
Triad	Major triad (r)	4	7	–
Triad	Major 3rd + octave	4	12	–
Triad	Suspended 4th	5	7	–
Triad	Major triad (2nd)	5	9	–
Triad	Power chord	5	12	–
Triad	Power chord	7	12	–
Triad	Minor 6th + octave	8	12	–
Triad	Major 6th + octave	9	12	–
Tetrad	Minor triad (r) + octave	3	7	12
Tetrad	Major triad (1) doubled 3rd	3	8	12
Tetrad	Major 7th chord	4	7	11
Tetrad	Major triad (r) + octave	4	7	12
Tetrad	Major 3rd + major 7th + octave	4	11	12
Tetrad	Suspended 4th + major 6th	5	7	9
Tetrad	Suspended 4th + octave	5	7	12
Tetrad	Major triad (2nd) doubled 3rd	5	8	12
Tetrad	Major triad (2) doubled 5th	5	9	12
Tetrad	Major triad (r) + major 2nd	2	4	7
Dissonant chords				
Dyad	Minor 2nd	1	–	–
Dyad	Major 7th	11	–	–
Triad	Minor 2nd + major 2nd	1	2	–
Triad	Minor 2nd + major 7th	1	11	–
Triad	Minor 3rd + major 3rd	3	4	–
Triad	Minor 4th + major 4th	5	6	–
Triad	Minor 6th + major 6th	8	9	–
Triad	Major 6th + minor 7th	9	10	–
Triad	Minor 7th + major 7th	10	11	–
Triad	Major 7th + octave	11	12	–
Triad	Minor 2nd + major 6th	1	9	–
Triad	Minor 2nd + minor 7th	1	10	–
Tetrad	Minor 2nd + major 2nd + octave	1	2	12
Tetrad	Minor 2nd + minor 3rd + minor 7th	1	3	10
Tetrad	Perfect fifth + minor 6th + major 6th	7	8	9
Tetrad	Minor 6th + major 6th + minor 7th	8	9	10
Tetrad	Minor 6th + minor 7th + major 7th	8	10	11
Tetrad	Major 6th + minor 7th + major 7th	9	10	11
Tetrad	Minor 2nd + major 6th + minor 7th	1	9	10
Tetrad	Minor 2nd + major 6th + major 7th	1	9	11
Tetrad	Minor 2nd + major 2nd + minor 7th	1	2	10
Tetrad	Minor 2nd + major 2nd + major 7th	1	2	11

Dyads, triads, and tetrads comprised two, three and four vocalizations, respectively. The “name” column refers to the naming of the chord or its composite intervals as defined by Western music theory. Numbers in the “tone2”, “tone3”, and “tone4” columns describe the number of semitones that each tone in a given chord was above lowest tone in the chord. These chords comprise the two most and least consonant dyads, the five most and least consonant triads, and the five most and least consonant tetrad, as assessed empirically in Bowling et al. 2018

#### Analysis

As the data could not be assumed to be normally distributed (Shapiro–Wilk, *p* = 0.02501), we used the Wilcoxon signed-rank test and the Friedman test in analysis parallel to that of the budgerigar results from Experiment 1.

### Results

One male budgerigar was excluded from analysis for not listening to both a consonant and a dissonant stimulus before reaching the cut-off point of six sessions (12 h).

**Fig. 5:**
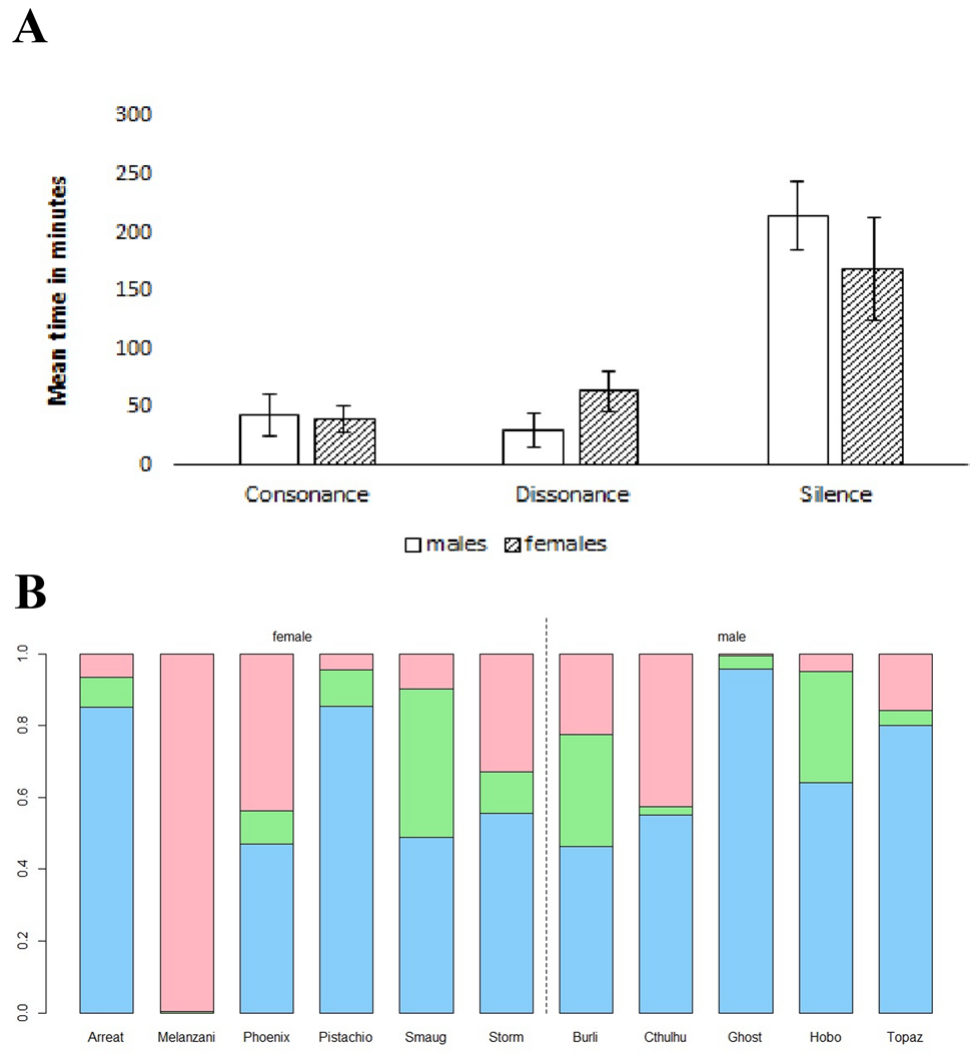
**a** Mean average time spent with consonant and dissonant stimuli for budgerigars separated for males and females. Error bars show standard error. **b** Relative time spent with consonant and dissonant stimuli for budgerigar subjects. Green is time on consonant (middle), red is time on dissonant (top), and blue is time on silent (bottom). Females are to the left of the dividing line, males are to the right (color figure online)

Figure 5a gives the mean average and standard error for time spent with the different stimulus types and silence for males and females, respectively. See Fig. 5b for a visual representation of relative time spent with consonant stimuli, dissonant stimuli and on neither side for individuals.

We used a Friedman test to compare relative time spent on the consonant (*M* = 41 min 4 s, SD = 37 min 36 s), dissonant (*M* = 48 min 13 s, SD = 40 min 13 s) and silent condition (*M* = 160 min 25 s, SD = 94 min 14 s). Budgerigars spent significantly more time in the silent condition than in the other conditions. (Friedman, *p* = 0.003905).

Additional Wilcoxon signed-rank tests analysing each sex separately showed that, for males, there was no significant difference in time spent with consonant (*M* = 42 min 46 s, SD = 39 min 16 s) or dissonant (*M* = 29 min 38 s, SD = 25 min 41 s) stimuli (Wilcoxon, *p* = 0.5938). The same is true for females: The difference between time spent with consonant (*M* = 39 min 38 s, SD = 36 min 6 s) and dissonant (*M* = 63 min 42 s, SD = 43 min 27 s) stimuli was found to be not significant (Wilcoxon, *p* = 0.7812).

To control for an effect of exposure, we also analysed data only from beyond the point where birds had heard each stimulus at least once. We found no diverging results with all *p* values > 0.05 (for all birds: Wilcoxon *p* = 0.5; for males only: Wilcoxon *p* = 0.5938; for females only: Wilcoxon *p* = 0.8125).

#### Summary

In comparison to Experiment 1, this experiment used more ecologically relevant stimuli built from budgerigar vocalizations with the intention of increasing the salience of stimuli for budgerigars to create a better test of whether budgerigars may exhibit consonance preferences. The results suggest that budgerigars preferred silence to both the consonant and the dissonant stimuli. This means that although our stimuli may have had the effect of increasing the salience of these stimuli for budgies, they did so in a negative way. It should be noted that this was not our intention and that it effectively precluded us from observing any potential effect of increasing stimulus salience on consonance preferences.

## Discussion

The basic logic underlying our experimental approach is that individual members of a species that harbors an attraction to consonance would be expected to spend more time in locations where consonant sounds are heard, relative to locations where dissonant sounds are heard. Therefore, the results of both experiments in this study suggest that budgerigars are not attracted to consonance over dissonance or vice versa.

Intriguingly, when a similar paradigm was applied to humans, only females spent more time in locations when they heard consonant sounds. Sex differences in humans’ reaction to musical stimuli are rarely tested but have been described (Bowling et al. 2019; Nater et al. 2006; Wuttke-Linnemann). Still, that males did not spend more time with consonant stimuli was surprising, as the traditional survey-based tests of consonance preferences for humans have not produced any evidence of sex differences of which we are aware. Accordingly, the survey ratings of the stimuli from Experiment 1 made by human subjects here suggest that the consonant stimuli were perceived as more preferable than the dissonant stimuli by both females and males, validating their use in the present study. Looking at the survey results more closely, the behaviour of females during the experiment was indeed related to their preference ratings, i.e., the greater the difference in female ratings of preference for consonant over dissonant stimuli, the more time they spent listening to them in the place preference experiment. However, while males also found consonant stimuli more preferable than dissonant stimuli, they rated the dissonant stimuli as more interesting than the consonant stimuli, whereas females did not. In line with this, the greater the difference in male ratings of interest for dissonant stimuli, the more time they spent listening to them in the place preference experiment. Considering that the stimuli in Experiment 1 consisted of a relatively simple melody repeated over and over, it is not hard to see how subjects may have found these stimuli less than interesting. Some research suggests that human males are more susceptible to boredom, less averse to novelty, and more prone to thrill seeking compared to females (Cross 2013; Kurtz 1978; McIntosh 2006), which may provide an explanation for the sex difference encountered with this paradigm. A follow-up study with humans could additionally try to disentangle confounds of interest in consonance and consonance preference, especially for males. To this end, simple stimuli like the ones used here could potentially be contrasted with more complex and variable ones. In any case, it is clear that further studies with larger sample sizes will be needed to conclude whether the novel result regarding a sex difference in human reaction to consonance found in this study is replicable or an artefact of the specific stimuli or setup used in this study.

Regarding the budgerigar results, it should be mentioned that there are of course numerous variations on the paradigm used here, the application of which may yet produce different results. One potential variation concerns species-specific stimuli. Previous research suggests that use of species-specific stimuli in auditory research is recommendable (Snowdon and Teie 2010). However, in the experiment using budgerigar-specific stimuli, the birds spent most time on the silent condition, which was not the case with piano stimuli. This could be connected to differences in auditory masking arising from background playback of the bird’s home colony during the experiments (designed to decrease stress resulting from being isolated from the colony). In particular, budgerigar-specific stimuli may have masked the colony playback (or vice-versa) more than the piano stimuli. Considering how highly social budgerigars are, the tendency to spend most time on silent, the condition where colony playback would have been most audible, would then be less surprising. Notably, however, we have not observed similar avoidance of stimuli comprised of budgie vocalizations in our other studies using this setup, although these stimuli were less harmonic overall (Afroozeh et al. in prep; Kopaç et al. in prep). Another, mutually non-exclusive, potential explanation for the silence preference of budgies in Experiment 2 is that they found the synthetic budgie vocalization chords to be exceptionally strange, in that they were quite similar to their own vocalizations but unnaturally arranged, possibly resulting in a sort of budgerigar “uncanny valley” effect. To our knowledge, nobody has yet found such an effect in birds, yet its existence appears possible: It has been suggested that budgerigars, like other species, have specializations in processing conspecific vocalizations allowing for finer discrimination of such sounds than of e.g. vocalizations by other species (Dooling et al. 1992). It is not entirely clear which specific properties of a sound are most important to these discriminations, though temporal cues seem to be less important than frequency bandwidth (Okanoya and Dooling 1991). Budgerigars have also been shown to be more sensitive to mistunings of single harmonics within complex sounds than humans are (Lohr and Dooling 1988), suggesting spectral distribution of energy—which was manipulated here—is perhaps especially salient to their hearing. Further research could tackle more directly the question of which properties of a sound are important for budgerigar conspecific call recognition (e.g. Beckers et al. 2003). Until then, the possibility that manipulation of the stimuli may have offset processes of conspecific call recognition in a way that was detrimental to the goals of this study remains. In sum, this result and its potential explanations may be of interest for researchers considering the advantages and disadvantages of using species-specific stimuli, as it appears that the manipulated species-specific calls used here were off-putting to our subjects. However, other methods of manipulation may be able to circumvent the issue described here. Such alternative methods could for example include constructing arpeggios from unaltered budgerigar vocalizations or shifting the harmonics within vocalizations to produce consonant or dissonant intervals, both of which would retain more of the natural structure of budgerigar vocalizations than the stimuli used here.

Another possibility for variation of our paradigm concerns development. One of the few successful demonstrations of an attraction to consonance in animals was confined to early development (Chiandetti and Vallortiagra 2011). Early influences may play an important role in development of an attraction to consonance. A study with newly hatched or young budgerigars bred and reared unter controlled circumstances could exclude that possibility. Additionally, conducting a study with young birds may also be worthwile as it could potentially show attraction to sounds in early developmental stages that may be more difficult to demonstrate for adults. Budgerigar’s hearing sensitivities change during early development (Brittain-Powell and Dooling 2004), and so may sound preferences. With regards to a potential connection of vocal learning and attraction to consonance, it should be mentioned that hearing abilities are adult-like when vocal learning abilities emerge at 4 weeks post fledgling (Brittain-Powell and Dooling 2004). These learning abilities are retained throughout budgerigar adult life (Farabaugh et al. 1994). However, the point of emergence may be ideal to test for attraction to certain sounds as such an attraction (perhaps leading to increased copying of those sounds) may potentially become less pronounced in later life by exposure to a variety of influences.

Apart from variations on the paradigm, future studies may also want to consider entirely different approaches. VSH posits that attraction to vocal sounds is connected to our attraction to consonance in general. However, in non-human animals, the context in which such an attraction is tested seems likely to be highly important. Although humans seem to enjoy listening to or otherwise engaging with music in a variety of contexts, animal engagement with musical stimuli (or stimuli with musical elements) may be more limited. For example, in the most robust demonstration of consonance preferences in a non-human animal, Chiandetti and Valloritgara (2011) focused on newly hatched chicks, suggesting that their perference to approach an object associated with consonant sounds reflects a specific developmental context in which they are driven to identify their mother, whose vocalizations would be distiguished from other environmental sounds by their highly harmonic structure. Whether or not the attraction to consonance that they demonstrated would generalize to other contexts or be retained into their adult life is unknown. As suggested in the introduction, one context in which an attraction to vocal sounds may be particularly beneficial to budgerigars is when they are trying to learn the vocalizations of their social group. In general, vocal learning animals may benefit from preferentially attending to harmonic sounds over inharmonic ones, as this would assist them in attending and processing the vocal sounds to be imitated. Thus, an extension of the present study focusing on budgerigars attempting to learn conspecific vocalizations may be a more effective approach to studying any potential consonance preferences. Budgerigars could be trained to mimic consonant and dissonant pitch sequences. Learning curves, number of imitated stimuli and the time needed to successfully imitate the different stimuli could serve as measurements for a potential preference in imitating either sort of stimuli (Manabe et al. 2008) and thereby apply a first direct test to the idea that attraction to consonance may be relevant to vocal learning.

It should also be noted that so far, no study has directly tested whether budgerigars perceive sounds as belonging to consonant and dissonant categories. Budgerigars have been shown to outperform humans at frequency discrimination tasks (Dent et al. 2000), to have highly accurate pitch perception (Weisman et al. 2004) and to outperform humans at detecting tuning alterations of single harmonics within complex sounds (Lohr and Dooling 1988). All of this strongly suggests that budgerigars are very likely to be capable of perceiving the differences between the consonant and dissonant stimuli used here. Still, future studies of attraction to consonance in budgerigars may want to test this ability before proceeding with tests of attraction towards consonance. Testing the ability to discriminate consonant and dissonant stimuli could be performed using operant discrimination tasks similar to the ones that have been successfully applied with Java sparrows (Watanabe et al. 2005) and European starlings (Hulse et al. 1995).

Considering all these potential variations and alternative directions, it is clear that making final conclusions about the absence of attraction to consonance in budgerigars would be premature. However, should future studies also fail to provide evidence for an attraction to consonance in budgerigars, this could be due to several reasons. While the degree to which human consonance preferences are determined by cultural traditions remains debated, and evidence from infant studies is equivocal (Masataka 2006; Perani et al. 2010; Schellenberg and Trehub 1996; Trainor and Heinmiller 1998; Trainor et al. 2002; Trehub 2003; Zentner and Kagan 1996, 1998; but see Platinga and Trehub 2014), the highly non-random structure of music and the extent to which it is preserved across cultures and throughout time still suggests important biological constraints (Bowling et al. 2017). This would suggest that differences in human and budgerigar biology could be the reason for differences in attraction to consonance and dissonance with crucial differences most probably occurring in vocal production. According to VSH, human consonance preference is connected to an attraction to the human voice (Bowling and Purves 2015; Bowling et al. 2018). If budgerigars do not exhibit attraction to consonance, this may be because of differences in their vocalizations, and/or the ways in which they use vocalizations to communicate. One such crucial difference may be the relative commonness of nonlinear phenomena in vocalization. Nonlinear phenomena in vocal production produce deterministic chaos where harmonic structure is masked by energy similar to (but unlike) turbulent noise (Fitch et al. 2002). This means that such sounds are less clearly harmonic. Importantly, in human adults, the use of such vocalizations is generally avoided in speech as in song (Fitch et al. 2002; Arnal et al. 2015). For budgerigar vocalizations, nonlinearity is relatively common, in warble as well as in frequently used and socially important contact calls (Lavenex 1999; Tu et al. 2011). There is a connection to VSH here: the almost exclusive use of clearly harmonic vocalizations and avoidance of nonlinear vocalizations in speech (the dominant mode of human auditory–vocal communication), may facilitate the development of attraction to harmonic sounds, and thereby to consonance in our species. On the other hand, Budgerigars’ routine use of less clearly harmonic, nonlinear vocalizations may conversely hinder the emergence of such an attraction. In addition to this purely physical connection, nonlinearity in mammals is usually associated with specific contexts, namely ones of duress (Blumstein et al. 2008; Gouzoules et al. 1984; Held et al. 2006), occurring frequently for example in screams, particularly of infants (Robb and Saxman 1988; Sirviö and Michelsson 1976; Truby and Lind 1965). In line with this, nonlinear vocalizations apparently increase tension and evoke fear in humans (Blumstein et al. 2010). This connection may additionally heighten an attraction to clearly harmonic sounds in mammals as they would be associated with more relaxed states. For budgerigars, an association of nonlinearity with more negative emotional states has not been documented, and the fact that nonlinearity occurs in contact calls would appear to speak against such a connection. Future studies testing species with more clearly harmonic vocalizations and less nonlinearity in vocal output may provide an interesting perspective on the idea of a connection between the commonness of nonlinear phenomena and the development of an attraction to consonance.

Additionally, differences in vocal communication may be relevant to understanding why budgerigars may not be attracted to consonance. Apart from VSH, reasons for humans’ heightened interest in consonance and dissonance may be found in the fact that humans are sexually dimorphic in terms of speaking pitch, and exhibit age-dependent pitch changes. The octave is generally regarded as the most consonant interval (see e.g. Bowling and Purves 2015; Bowling et al. 2018; Burns 1999) and used in imitation of other human voices: humans tend to imitate vocal pitches outside their own vocal range by shifting fundamental frequency by an octave (Brown and Jordania 2013), a behaviour that is related to perception of octave equivalence (Peter et al. 2008, 2009, 2015). These connections may provide one reason, why intervals that are pleasurable to humans appear not to be of heightened interest for budgerigars. Budgerigars are not sexually dimorphic in their vocal ranges (both male and female approximately 1000–5700 Hz; Brittain-Powell et al. 2002; Farabaugh et al. 1998) and while they do have highly accurate pitch perception (Weisman et al. 2004) they do not appear to perceive octave equivalence but rather seem to group tones in a different (but non-random) way (Hoeschele et al. 2012; Wagner et al. 2019). While the mechanics and possible pattern of this grouping remain unclear (Hoeschele et al. 2013; Wagner et al. 2019), the evidence suggests important differences in the way humans and budgerigars perceive tones that could be directly relevant to understanding potential inter-specific differences in consonance perception.

Future research may also wish to investigate questions of phylogeny of attraction to consonance in birds. As stated above, several songbird species preferentially produce harmonic intervals (Doolittle and Brumm 2012; Doolittle et al. 2014; Richner 2016), but experimentally testing such species for an attraction to consonance remains to be performed. Beyond chickens and the psittacopasserean parrots and songbirds (Suh et al. 2011), species from other groups could also be tested to shed light on the question how widespread attraction to consonance is among avians. Pigeons spring to mind as an ideal choice for having been established as model organisms in cognitive experiments and shown to be able to discriminate between some musical stimuli (Brooks and Cook 2009; Porter and Neuringer 1984). In the short term, however, any study that tests non-human animal species for attraction to consonance will help in generating a better understanding of the prevalence and potential evolutionary functions of the phenomenon of attraction to consonance. For these purposes, the methodological challenges described here, and ways of overcoming them, will be critical to future research.

In conclusion, this study represents the first experimental test for an attraction to consonance in a vocal learning bird species, and only the second to examine consonance in a bird species in general. More comparative research in this area is needed, because similarities and differences in auditory–vocal communication between humans and other species can be leveraged to understand the biological factors that underlie the nature of auditory perception. Variations on the paradigm here can be useful in future consonance studies with budgerigars as well as other species. While we will continue our research in this direction, we actively invite other researchers to further investigate consonance—a hallmark of human music—in a greater variety of species.
